# Predictive Value of *STC2* Gene Expression in Chemotherapy Response in Breast Cancer

**DOI:** 10.3390/ph18020235

**Published:** 2025-02-08

**Authors:** Juan P. Muñoz, Nicolás Lampe-Huenul

**Affiliations:** Laboratorio de Bioquímica, Departamento de Química, Facultad de Ciencias, Universidad de Tarapacá, Arica 1000007, Chile

**Keywords:** stanniocalcin-2 (STC2), breast cancer, doxorubicin, chemotherapy resistance, predictive biomarker, cancer prognosis

## Abstract

**Background**: Breast cancer is the most commonly diagnosed cancer among women, and resistance to chemotherapy presents a significant challenge in its treatment. Stanniocalcin-2 (STC2), a glycoprotein involved in calcium homeostasis and cellular stress responses, is frequently overexpressed in various human cancers. Despite its critical role in cellular adaptation to stress, the potential of *STC2* as a biomarker for predicting chemotherapy response has not been evaluated. This study aimed to assess the potential of *STC2* as a predictive biomarker of response to chemotherapy in breast cancer. **Methods**: We utilized publicly available databases to characterize *STC2* expression in breast cancer patients and its role in predicting relapse-free survival (RFS). Moreover, we evaluated the treatment responses of patients subjected to chemotherapy, correlating their outcomes with *STC2* expression levels to determine its potential as a predictive biomarker. Finally, we evaluated the *STC2* expression levels in breast cancer cell lines following exposure to doxorubicin (Dox), the primary anthracycline used in chemotherapy, and they were contrasted with the publicly available dataset. **Results**: The analysis showed that *STC2* is significantly overexpressed in luminal A breast cancer, where it is linked to genetic amplifications. High *STC2* expression was associated with improved RFS in ER-positive patients but correlated with worse outcomes in ER-negative cases. Furthermore, in grade II ER-positive patients, higher STC2 expression is linked to better chemotherapy response, while in grade II ER-negative patients, it was associated with poorer response. Finally, *STC2* downregulation was observed in response to Dox treatment. **Conclusions**: These findings suggest that *STC2* expression serves as a predictive biomarker for chemotherapy response in grade II breast cancer patients.

## 1. Introduction

Stanniocalcin-2 (STC2) is a member of a conserved family of secreted glycoprotein hormones originally identified in fish, where it plays a role in regulating calcium levels [1]. In humans, the *STC2* gene is located on chromosome 5q35.1, and it is widely expressed in various tissues, including the breast, muscle, heart, and pancreas [2]. *STC2* has been shown to participate in numerous physiological processes, such as calcium and phosphate homeostasis, cellular stress response, cellular metabolism, inflammation, and antioxidative function [3,4,5,6,7,8].

Recent studies have highlighted *STC2* as a gene of interest in cancer biology due to its upregulation in a wide range of human cancers, including breast cancer, esophageal squamous-cell carcinoma, hepatocellular carcinoma, colorectal cancer, gastric cancer, renal cell carcinoma, and prostate cancer [9,10,11,12,13]. Interestingly, the expression of *STC2* is closely associated with tumor progression and metastasis [14]. In breast cancer specifically, *STC2* is frequently co-expressed with the estrogen receptor (ER) [15], a key driver of tumor growth expressed in about 80% of breast cancer cases [16]. In fact, *STC2* expression is positively regulated by estrogen signaling, and higher levels of *STC2* have been linked to better disease-free survival rates in ER-positive breast cancer patients. However, this correlation does not hold true for hormone receptor-negative patients [17]. Moreover, recent studies have shown that STC2 suppresses the migration and invasion of triple-negative breast cancer by inhibiting epithelial-mesenchymal transition (EMT) and promoting cell apoptosis [18].

One of the primary challenges in breast cancer treatment is overcoming resistance to chemotherapeutic compounds, particularly anthracyclines and taxanes [19]. Doxorubicin (Dox) is an anthracycline-class anticancer agent widely utilized in the treatment of various human malignancies, including leukemias, neuroblastoma, bone sarcomas, ovarian cancer, thyroid cancer, gastric cancer, lymphoma, lung cancer, and breast cancer [20]. Dox works by intercalating into DNA and inhibiting topoisomerase II, leading to DNA damage and cell death [21]. However, many patients develop resistance, which limits the effectiveness of the drug and leads to cancer recurrence [22,23].

Under stress conditions, including endoplasmic reticulum stress, hypoxia, and nutrient deprivation, *STC2* expression is significantly upregulated, promoting the maintenance of redox homeostasis, resistance to apoptosis, and cell survival [7,24,25]. These stress-related functions of *STC2* are especially pertinent within the tumor microenvironment, where cancer cells frequently encounter adverse conditions that foster the selection of more aggressive and therapy-resistant cell populations [26]. Despite its critical role in cellular adaptation to stress, the potential of *STC2* as a biomarker for predicting chemotherapy response has not yet been evaluated.

Given the critical role of *STC2* in the stress response, the aim of this work is to evaluate the potential of *STC2* as a predictive biomarker of response to chemotherapeutic agents in breast cancer. To this end, we evaluated *STC2* expression patterns from public databases and their impact on treatment outcomes. The results shown here provide evidence that *STC2* gene expression could serve as a predictive biomarker for chemotherapy response in breast cancer patients.

## 2. Results

### 2.1. STC2 Is Overexpressed in Luminal A Breast Cancer

**Figure 1:**
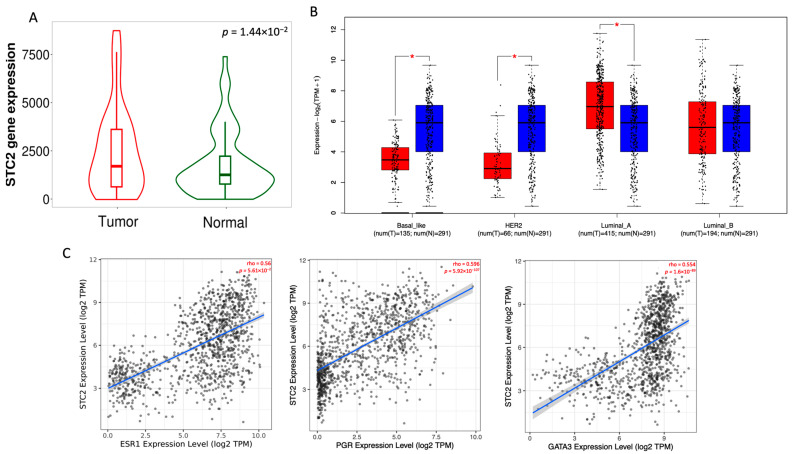
*STC2* expression analysis in breast cancer tissues compared to normal tissues. (**A**) Violin plot showing *STC2* gene expression levels in normal and tumor tissues. The plot includes paired tumor and adjacent normal tissues (**B**) Box plots showing *STC2* expression in different breast cancer subtypes. Tumor samples are shown in red; Normal samples in blue. (**C**) Correlation plots showing the association of *STC2* expression with luminal markers *ESR1*, *PGR*, and *GATA3*. Each plot displays the correlation coefficient (rho) and *p*-value, indicating significant positive correlations between *STC2* and the luminal markers. * *p* < 0.05.

To assess the expression of the *STC2* gene across multiple cancer types, we first analyzed *STC2* mRNA expression levels in tumor and normal tissues using the TNM platform, which integrates data from Gene Expression Omnibus (GEO), Genotype-Tissue Expression (GTex), The Cancer Genome Atlas (TCGA), and Therapeutically Applicable Research to Generate Effective Treatments (TARGET) databases. As shown in Figure S1, *STC2* is overexpressed across a range of cancers, including bladder, colon, esophagus, liver, lung, ovary, pancreas, prostate, rectum, renal cell carcinoma, skin, testis, thyroid, and uterus. Notably, *STC2* mRNA in breast cancer is significantly elevated compared to their corresponding normal tissues (*p* = 1.44 × 10^−2^, Figure 1A). The analysis revealed that all tumors have *STC2* expression above the minimum cutoff. The percentage of tumors exceeding higher cutoffs decreases progressively, with 74.3% above Q1, 65.7% above the median, 34.3% above Q3, and only 1.4% above the maximum cutoff (Figure S2). This pattern suggests a widespread but variable overexpression of *STC2* in breast cancer cases. Interestingly, *STC2* overexpression in breast cancer is strongly associated with genetic alterations, such as amplifications and gains, which could explain its overexpression (Figure S3).

To further explore the expression patterns of *STC2* across different molecular subtypes of breast cancer, we analyzed its expression using the Gene Expression Profiling Interactive Analysis 2.0 (GEPIA2.0) platform. Our analysis revealed that *STC2* is predominantly overexpressed in the luminal A subtype, while other subtypes exhibit significantly lower or non-significant expression levels (Figure 1B).

To confirm this finding, we performed correlation analyses between *STC2* and key luminal A markers, including *ESR1*, *PGR*, and *GATA3*, using the TIMER2.0 platform. As shown in Figure 1C, we observed strong positive correlations, with Spearman’s coefficients of 0.56, 0.596, and 0.554 for *ESR1, PGR*, and *GATA3*, respectively, all with highly significant *p*-values (*p* < 0.001).

### 2.2. STC2 Is Associated with ER Regulation

**Figure 2:**
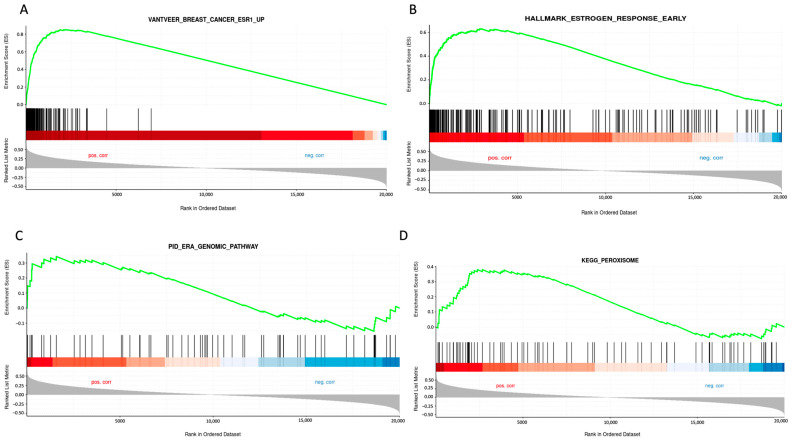
GSEA of *STC2* expression in breast invasive carcinoma using GENI web server. The analysis was conducted by selecting breast tissue and focusing on *STC2* from the TCGA PanCancer Atlas dataset. Predefined Curated (**A**), Hallmark (**B**), PID (**C**), and KEGG (**D**) gene sets were applied. The colors in the gradient below the enrichment curve reflect the correlation with the studied condition: the red area indicates genes positively correlated with *STC2* expression in breast invasive carcinoma, while the blue area represents genes with a negative correlation.

To further elucidate the role of *STC2* in luminal A breast cancer, we performed gene set enrichment analysis (GSEA) on breast cancer samples from the TCGA database, focusing on cases with high *STC2* expression. This analysis showed a significant enrichment of gene sets related to ER regulation and associated pathways, indicating a strong link between *STC2* and ER-driven breast cancer. Notably, the analysis highlights enrichment in gene sets such as “Vantveer_Breast_cancer_ESR1_Up” and “Hallmarck_Estrogen_Response_Early”, suggesting that *STC2* expression is positively correlated with genes upregulated in ER-positive breast cancer and early estrogen response (Figure 2). Additionally, the “KEGG_Persoxisome” pathway was also enriched, indicating a potential role of *STC2* in peroxisome-related processes linked to cellular metabolism and oxidative stress. Collectively, these findings support the hypothesis that *STC2* plays a critical role in ER-positive breast cancer by modulating estrogen signaling and peroxisome-related processes.

### 2.3. Prognostic Impact of STC2 in Breast Cancer Is Dependent on ER Status

**Figure 3:**
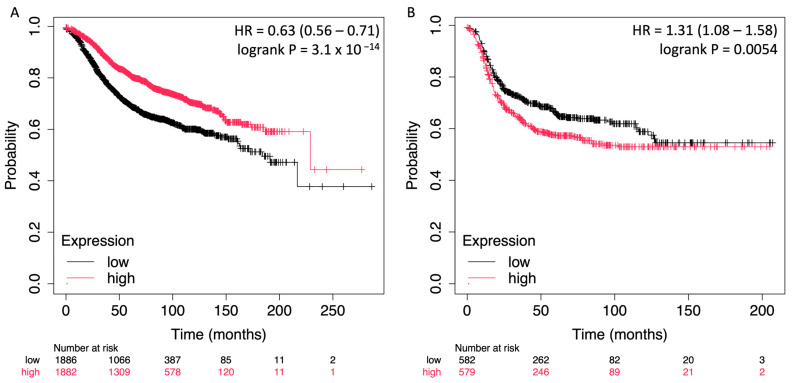
Kaplan–Meier survival curves showing the impact of *STC2* expression on RFS in ER-positive (**A**) and ER-negative (**B**) breast cancer patients. The curves depict the probability of RFS over time for each group, with high *STC2* expression denoted in red and low *STC2* expression in black.

To assess the prognostic value of *STC2* in relation to ER expression in breast cancer, survival analyses were conducted using the Kaplan–Meier plotter platform [27]. The analyses were stratified by ER status, allowing for a comparison between ER-positive and ER-negative breast cancer patients, each of whom was followed for relapse-free survival (RFS). For the ER-positive cohort, the results indicated a significant association between high *STC2* expression and improved survival outcomes. The hazard ratio (HR) for high vs. low expression was 0.63 (95% CI: 0.56–0.71), with a highly significant log-rank *p*-value of 3.1 × 10^−14^. The Kaplan–Meier survival curve shows a clear separation between the high and low *STC2* expression groups, with patients in the high-expression group demonstrating substantially better RFS across the follow-up period. (Figure 3A). Conversely, in the ER-negative cohort, high *STC2* expression was associated with worse survival outcomes. The hazard ratio for high vs. low expression was 1.31 (95% CI: 1.08–1.58), and the log-rank *p*-value was 0.0054, indicating a significant correlation. The survival curve demonstrates that patients with higher *STC2* expression had a lower probability of RFS compared to those with lower expression, suggesting that *STC2* has an adverse prognostic role in ER-negative breast cancer.

### 2.4. Differential STC2 Expression as a Predictor of Chemotherapy Response in ER-Positive and ER-Negative Breast Cancer Patients

**Figure 4:**
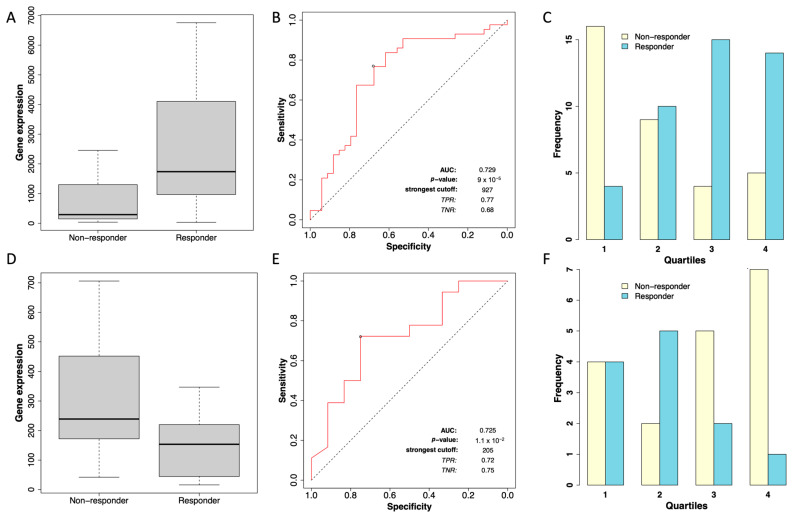
Expression of *STC2* and its association with chemotherapy response in grade II breast cancer patients. (**A**) Box plot showing *STC2* expression levels comparing non-responders and responders to chemotherapy in grade II ER-positive breast cancer patients. (**B**) ROC curve evaluating the diagnostic performance of *STC2* expression in ER-positive breast cancer patients. (**C**) Distribution of response status across quartiles of *STC2* expression in ER-positive breast cancer patients. (**D**) Box plot comparing *STC2* expression levels between non-responders and responders to chemotherapy in grade II ER-negative breast cancer patients. (**E**) ROC curve assessing the predictive value of *STC2* expression for chemotherapy response in ER-negative breast cancer patients. (**F**) Distribution of response status across *STC2* expression quartiles in ER-negative breast cancer patients.

To evaluate the relationship between *STC2* expression and its association with RFS in patients treated with chemotherapy, we used the ROC Plotter server, a database designed to identify predictive biomarkers based on gene expression using transcriptomic data from a large cohort of breast cancer patients [28]. In this analysis, we observed that within the cohort of grade II ER-positive breast cancer patients (consisting of 43 responders and 34 non-responders), *STC2* expression showed a significantly higher expression in responders, with a fold change of 1.9 between both groups (Figure 4A). The Mann–Whitney test confirmed that this difference in expression levels was statistically significant (*p* = 4.7 × 10^−3^). The receiver operator characteristic (ROC) curve analysis yielded an area under the curve (AUC) of 0.729 (*p* = 9 × 10^−5^), indicating an acceptable predictive value of *STC2* for chemotherapy response in this subgroup (Figure 4B). The distribution of *STC2* expression across quartiles (Figure 4C) further highlights a higher frequency of responders in the higher *STC2* expression quartiles, supporting the observed trend.

In the grade II ER-negative cohort, the analysis included 12 responders and 18 non-responders. In contrast to the ER-positive group, responders in the ER-negative cohort exhibited reduced expression of *STC2*, with a median expression of 154 in responders compared to 239 in non-responders (fold change of −1.9 between both groups, Figure 4D), suggesting an inverse relationship between *STC2* levels and chemotherapy response in grade II ER-negative patients. The Mann–Whitney test showed that this reduction was statistically significant (*p*-value = 0.042). The ROC curve analysis for the ER-negative cohort yielded an AUC of 0.725, suggesting an acceptable discriminatory power (Figure 4E). Additionally, the distribution of *STC2* expression across quartiles in grade II ER-negative breast cancer patients shows a contrasting pattern to that of the ER-positive cohort (Figure 4F). Although the predictive value of *STC2* remains moderate, the pattern of reduced expression in responders suggests a different biological role of *STC2* in ER-negative patients compared to ER-positive cases. In grade I or III breast cancer patients, no statistically significant differences were observed between responders and non-responders in either the ER-positive or ER-negative groups.

### 2.5. Dox Suppress STC2 Expression in Breast Cancer Cells

**Figure 5:**
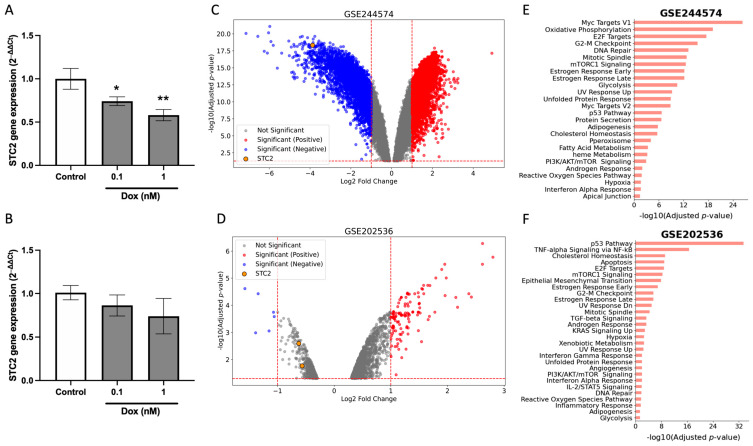
Dox induces a suppression of *STC2* gene expression. (**A**,**B**) RT-qPCR analysis of *STC2* gene expression in MCF7 (**A**) and MDA-MB-231 (**B**) cells exposed to Dox for 24 h. (**C**,**D**) Volcano plots of datasets GSE244574 and GSE202536 showing differentially upregulated (red) and downregulated (blue) genes, with *STC2* highlighted in a yellow dot. (**E**,**F**) Bar charts represent the top enriched pathways based on adjusted *p*-values. Data are presented as mean ± standard error of the mean from triplicate experiments. (* *p* < 0.05, ** *p* < 0.01).

In order to evaluate the underlying mechanisms driving the differential chemotherapy response observed between ER-positive and ER-negative cohorts, we examined the effects of Dox on *STC2* expression in breast cancer cell lines. We conducted RT-qPCR experiments on the ER-positive MCF7 and ER-negative MDA-MB-231 cell lines following 24-h Dox treatment. The results revealed a significant downregulation of *STC2* mRNA expression in MCF7 cells (Figure 5A), while no significant changes were observed in MDA-MB-231 cells (Figure 5B).

To validate these findings, we analyzed two publicly available transcriptome datasets, GSE244574 [29] and GSE202536 [30], which examined the response to Dox treatment in MCF7 and Cal51 breast cancer cell lines, respectively. Differential expression analysis revealed significant alterations in gene expression following Dox exposure. In the GSE244574 dataset (MCF7 cells), 4302 genes were upregulated, while 4183 genes were downregulated after Dox treatment (Figure 5C). Similarly, in the GSE202536 dataset (Cal51 cells), comparing untreated cells with those exposed to Dox for 24 h, 1067 genes were upregulated, and 268 genes were downregulated (Figure 5D). Functional enrichment analysis of the differentially expressed genes (DEGs) indicated that in MCF7 cells, the DEGs were predominantly associated with Myc and E2F targets, oxidative phosphorylation, G2-M targets, and DNA repair (Figure 5E). In contrast, the DEGs in Cal51 cells were primarily linked to the p53 pathway, TNF-alpha signaling, cholesterol homeostasis, and apoptosis (Figure 5F). Regarding *STC2* expression, both datasets demonstrated a statistically significant reduction in *STC2* levels following 24 h of Dox exposure (Figure S4). However, the extent of downregulation varied between the two datasets. In the MCF7 cells (GSE244574 dataset, Figure 5C), the downregulation of *STC2* was particularly robust, with a log2 fold change of −3.9 (*p* adj. = 7.37 × 10^−18^, *p*-value = 1.38 × 10^−20^). In contrast, while the GSE202536 dataset also showed statistically significant downregulation (*p* adj. = 2.54 × 10^−3^, *p*-value = 2.02 × 10^−5^), the change in *STC2* expression was moderate, with a log2 fold change of −0.62, representing a less pronounced decrease.

Finally, we analyzed *STC2* gene expression using the GSE240671 dataset [31], which contains RNA sequencing data from breast cancer patients before and after neoadjuvant chemotherapy. From the available data, we selected samples from three patients who underwent chemotherapy and had residual tumors following treatment. Our analysis showed a consistent downregulation of *STC2* gene expression in all three patients after neoadjuvant chemotherapy (Figure S5). The statistical analysis from the three samples confirmed these findings, revealing a log2 fold change of −1.39 with a *p*-value of 5.29 × 10^−3^.

Therefore, these results suggest that chemotherapy compounds lead to downregulation of *STC2* gene expression in both cellular models and clinical samples.

## 3. Discussion

In breast cancer treatment, chemotherapy remains a cornerstone of breast cancer treatment due to its effectiveness in targeting cancer cells. However, patient responses to chemotherapy are highly variable, influenced by factors such as genetic background, tumor subtype, and the presence of specific biomarkers that affect drug resistance and overall treatment outcomes [32].

This study highlights the complex regulatory mechanisms that cancer cells use to adapt to chemotherapy-induced stress, with a specific focus on STC2, a glycoprotein upregulated under various stress conditions, including hypoxia, endoplasmic reticulum stress, and nutrient deprivation, which are common in tumor microenvironment [33]. The results suggest that *STC2* is significantly overexpressed in breast cancer, especially in the luminal A subtype. This overexpression is closely linked to genetic alterations, such as gene amplifications and copy number gains, and it is associated with hormone receptor signaling pathways that are characteristic of luminal A tumors. Amplification of regions encoding *STC2* and its regulatory elements may drive its upregulation, suggesting a genetic basis for its elevated expression. Given its connection to hormone receptor signaling, *STC2* appears to play a pivotal role within ER pathways, which are central to the development and progression of luminal A tumors.

Since *STC2* is associated with ER signaling pathway as well as previous studies identifying *STC2* as a potential predictive marker for survival [34], we further analyzed its impact on RFS in breast cancer patients stratified by ER status. Using data from GEO and TCGA, we employed the Kaplan–Meier plotter, which revealed that *STC2* prognostic significance in breast cancer is dependent on ER status: The results showed that high *STC2* expression was associated with favorable outcomes in ER-positive cases but was linked to poorer prognosis in ER-negative cases. This differential prognostic impact of *STC2* expression in breast cancers could be explained by its interaction with ER-mediated signaling pathways. In ER-positive tumors, high *STC2* expression may enhance hormone receptor signaling, promoting cell differentiation and growth in a more regulated, less aggressive manner, therefore leading to improved treatment responses and favorable outcomes. Conversely, in ER-negative tumors, high *STC2* expression may activate alternative stress-response pathways, such as those related to hypoxia, nutrient deprivation, or endoplasmic reticulum stress, which could contribute to a more aggressive tumor phenotype and poorer prognosis.

In addition to these results, we demonstrated that *STC2* expression serves as a potential predictive marker for chemotherapy response in both ER-positive and ER-negative grade II breast cancer patients, but with different outcomes: In grade II ER-positive patients, higher *STC2* expression correlates with better chemotherapy response. In grade II ER-negative patients, higher *STC2* expression is associated with an unfavorable response to chemotherapy. The context-dependent role of *STC2* in breast cancer could explain its differential predictive value for chemotherapy response. In ER-positive tumors, high *STC2* expression likely interacts with ER-mediated pathways, reinforcing a more regulated and organized response to chemotherapeutic stress. This interaction may facilitate tumor cell susceptibility to chemotherapy by promoting apoptotic pathways or enhancing DNA damage response mechanisms specific to ER-positive cells, therefore leading to a better treatment outcome. In ER-negative tumors, however, *STC2* likely shifts to activating alternative stress-response mechanisms, such as those related to cellular adaptation to hypoxia, nutrient deprivation, or endoplasmic reticulum stress. These mechanisms could enable cancer cells to survive under the harsh conditions imposed by chemotherapy, contributing to chemoresistance. In this context, *STC2* overexpression may support cell survival by upregulating pathways that protect the tumor from the cytotoxic effects of treatment, leading to a poor chemotherapy response.

While anticancer therapies often induce a wide array of stress-related proteins, including heat shock proteins, RNA chaperones, and endoplasmic reticulum-associated stress proteins [35], our findings revealed downregulation of *STC2* in response to Dox treatment. This is contrary to the typical stress response, where stress-responsive genes are often upregulated, suggesting that *STC2* may be regulated by multiple cellular pathways activated by oxidative stress. It is well-documented that oxidative stress can repress gene expression even at non-cytotoxic levels, which may explain the observed downregulation of *STC2* [36,37,38]. In fact, in other models have been demonstrated that oxidative stress induces a downregulation of *STC2* [39].

On the other hand, Dox treatment is known to interfere with the activity of key transcription factors involved in various cellular processes [40,41]. This interference can occur through alterations in intracellular signaling pathways or direct post-translational modifications of transcription factors, reducing their capacity to promote gene expression. Furthermore, oxidative stress induced by Dox can impair ER function, altering the expression of ER-regulated genes [42]. Given that our data and previous reports suggest *STC2* is an estrogen-regulated protein [43], it is plausible that Dox disrupts the ER signaling pathways that typically drive *STC2* overexpression in cancer cells, leading to the downregulation of estrogen-responsive genes, including *STC2*.

The significant suppression of *STC2* expression following Dox treatment suggests that the drug may counteract the protective effects of *STC2*, therefore sensitizing cancer cells to apoptosis. This downregulation of *STC2* supports the hypothesis that *STC2* may contribute to chemoresistance in cancer therapy [44]. However, it is important to recognize that the datasets used in these analyses are not directly comparable due to several factors. For instance, the datasets were generated using distinct microarray platforms (Agilent and Affymetrix arrays), which can introduce variability in gene detection sensitivity, specificity, and expression quantification. Furthermore, differences in experimental conditions, sample handling, and platform-specific biases can lead to batch effects. Notably, the Dox concentrations varied between experiments, precluding a direct comparison of results. However, despite these limitations, the consistent observation of *STC2* downregulation upon Dox treatment across independent studies underscores the robustness of these findings and suggests a critical role for *STC2* in modulating the chemotherapeutic response. This warrants further investigation into *STC2* as a potential therapeutic target for overcoming chemoresistance.

The identification of *STC2* as a potential biomarker for predicting chemotherapy response has significant implications for breast cancer treatment. The ability to predict which patients are likely to respond to chemotherapy could guide personalized treatment strategies, potentially improving outcomes for those at risk of chemoresistance. Specifically, the stronger predictive value of *STC2* in ER-positive patients suggests that targeting *STC2* could be particularly beneficial in this subgroup.

Future studies should aim to validate these findings in larger and more diverse patient cohorts to confirm the utility of *STC2* as a biomarker for chemotherapy response. Additionally, mechanistic studies are needed to elucidate the molecular pathways through which *STC2* contributes to chemoresistance, particularly in the context of ER signaling and stress-response pathways. Understanding these mechanisms could provide insights into how *STC2* inhibitors might be developed and integrated into breast cancer treatment regimens. Furthermore, investigating the role of *STC2* in other subtypes of breast cancer, including triple-negative and HER2-positive cancers, could determine whether the findings are broadly applicable across breast cancer subtypes or specific to luminal A and ER-positive patients.

## 4. Materials and Methods

### 4.1. Cell Culture

Human breast cancer cell lines MCF7 (#C0006008) and MDA-MB-231 (#C0006002) were obtained from Addexbio (San Diego, CA, USA). MCF7 cells were cultured in Dulbecco’s Modified Eagle’s Medium (DMEM) (Gibco, Carlsbad, CA, USA) with the addition of 10 µg/mL insulin (Santa Cruz Biotechnology Inc., Santa Cruz, CA, USA) and 10% (*v*/*v*) fetal bovine serum (FBS) (Hyclone, Fremont, CA, USA). The MDA-MB-231 cells were grown in high-glucose DMEM (Gibco, Carlsbad, CA, USA) supplemented with 10% (*v*/*v*) FBS (Hyclone, Fremont, CA, USA). All cell lines were cultured at 37 °C in a humidified cell incubator with 5% CO_2_.

### 4.2. Real-Time Quantitative Reverse Transcription PCR (RT-qPCR)

Total RNA was extracted from the cultured cells using TRIzol Reagent (Invitrogen, Waltham, MA, USA), following the manufacturer’s instructions. Briefly, cells were lysed directly in TRIzol, and the homogenate was subjected to phase separation with chloroform (Merck, Darmstadt, Germany). The aqueous phase containing RNA was carefully transferred, and RNA was precipitated with isopropanol (Merck, Darmstadt, Germany). The RNA pellet was washed with 75% ethanol, briefly air-dried, and resuspended in RNase-free water. RNA concentrations were measured using the Qubit RNA BR assay kit and the Qubit 4 fluorometer (Thermo Fisher, Waltham, MA, USA). cDNA was synthesized from 1 µg of total RNA using the AffinityScript qPCR cDNA Synthesis Kit (Agilent Technologies, Inc., Santa Clara, CA, USA) according to the manufacturer’s protocol. The cDNA synthesis reaction was carried out in a 20 µL reaction volume, containing 10 µL of 2× master mix, 0.1 µg/µL of Oligo dT, and 1 µL of AffinityScript RT/RNase Block enzyme mix, and incubated at 37 °C for 30 min. For RT-qPCR, reactions were set up in a final volume of 25 µL, including 12.5 µL of Brilliant II SYBR Green q-RT-PCR 1-Step Master Mix (Agilent Technologies, Inc., Santa Clara, CA, USA), 0.5 µL of each primer (400 nM), 10.5 µL of nuclease-free water, and 1 µL of cDNA template. The primers used were: *β-actin* forward: TGCCGACAGGATGCAGAAG, *β-actin* reverse: GCCGATCCACACGGAGTACT; *STC2* forward: TCTTGTGAGATTCGGGGCTT, *STC2* reverse: ACAGGTCGTGCTTGAGGTAG. The *STC2* sequences were obtained from Huang et al. (2021) [45]. RT-qPCR was conducted on a CFX-96 real-time system (Bio-Rad Laboratories, Inc., Hercules, CA, USA) under the following cycling conditions: 94 °C for 30 s, 60 °C for 20 s, and 72 °C for 20 s, repeated for 40 cycles. All RT-qPCR assays were performed in triplicate, and β-actin was used as a reference gene for normalization. The relative expression levels were calculated using the 2^−ΔΔCt^ method.

### 4.3. Database Analysis

In this study, *STC2* mRNA expression in several cancers and corresponding normal tissues was examined using GEPIA2.0 plattform (http://gepia.cancer-pku.cn/; accessed on 10 July 2024) [46] and TNM web server (https://tnmplot.com/analysis/; accessed on 14 July 2024) [47]. Correlation analyses were conducted using the TIMER2.0 web server. (http://timer.cistrome.org/; accessed on 25 July 2024) [48,49,50]. These platforms facilitate the gene expression quantification using tumor and normal samples from TCGA and the GTEx databases. 

### 4.4. Datasets

The GEO database contains a vast repository of sequencing and microarray data contributed by research institutions worldwide. For this study, we searched datasets related to breast cancer and Dox exposure using the keywords “Breast cancer”, “Dox”, and “Chemotherapy”. This search resulted in the identification of the datasets GSE244574, GSE202536 and GSE240671. The GSE244574 dataset, published on 27 March 2024, by Naso et al. [29], provides transcriptomic data from Dox-treated MCF7 breast cancer cells and was generated using the Agilent-072363 SurePrint G3 Human GE v3 8x60K Microarray platform (039494). The GSE202536 dataset, published on 27 February 2023, by Kumar et al. [30], utilized the [HG-U133_Plus_2] Affymetrix Human Genome U133 Plus 2.0 Array (GPL570) in order to evaluate the differences between long-term resistance and short-term stress responses in triple-negative breast cancer cells. Specifically, in this database, we selected the data from Cal51 cells untreated and exposed to Dox for 24 h. Finally, we utilized the dataset GSE240671, published on 28 February 2024, by Derouane et al. [31]. This dataset, generated using the Illumina NovaSeq 6000 platform (GPL24676), includes RNA sequencing data from human mammary tumors collected from patients both before and after neoadjuvant chemotherapy. From the available data, we selected samples from three patients (R08, R13, and P56) who underwent neoadjuvant chemotherapy and had residual tumors after treatment. The inclusion criteria for this analysis were female patients aged 38 to 57 years at diagnosis, with breast cancer subtypes classified as either luminal B or triple-negative breast cancer (TNBC). All selected patients exhibited moderate or minimal residual disease post-treatment, and their molecular subtypes remained consistent both before and after chemotherapy.

### 4.5. Identification of DEGs

DEGs were identified using Python (v3.12.5) within a Jupyter Notebook environment. Key libraries employed in the analysis included GSEApy (v1.1.3) for gene set enrichment analysis. Standard libraries such as matplotlib (v3.9.2) were used for plotting, pandas (v2.2.2) for data manipulation, and numpy (v2.1.1) for numerical computations. DEGs were defined as genes with adjusted *p*-values less than 0.05 and |logFC| > 1. Volcano plots were generated using matplotlib to visualize the DEGs.

### 4.6. Functional Enrichment Analysis of DEGs

Functional annotation of DEGs was conducted using the GSEApy Python package, which supports GSEA and acts as a wrapper for Enrichr. This package enables easy enrichment analysis for Gene Ontology (GO) and KEGG pathways, producing high-quality figures. Lists of differentially expressed genes were provided for pathway enrichment, leveraging the statistical methods in GSEApy to identify significant biological processes and pathways. Additionally, GSEA analyses were performed using the GENI web server (https://www.shaullab.com/geni; accessed on 18 August 2024) from breast invasive carcinoma (TCGA, PanCancer Atlas) [51].

### 4.7. Survival Data Analysis

Survival data were analyzed using the Kaplan–Meier Plotter [27], focusing on RFS in breast cancer patients. Patients were divided into low and high *STC2* expression groups based on a 50% cutoff for both expression levels. Two separate analyses were performed: one for ER-positive and another for ER-negative breast cancer patients. The ER-positive cohort included 1372 patients (Affy ID: 203438_at), while the ER-negative cohort comprised 243 patients. Quality control steps, such as excluding redundant samples and removing biased arrays, were implemented to ensure the integrity of the data. Survival differences between the low and high *STC2* expression groups were assessed using the log-rank test, and hazard ratios (HR) with corresponding confidence intervals were calculated to quantify the risk. Additionally, ROC analyses were conducted using ROC Plotter [28] to evaluate the predictive value of *STC2* expression in chemotherapy-treated subgroups. The analyses were performed separately for ER-positive and ER-negative breast cancer patients, with responses based on 5-year RFS and grade II tumors. The predictive performance of *STC2* was determined by plotting ROC curves, and AUC values were calculated. The statistical significance of the differences between responders and non-responders was assessed using the Mann–Whitney U test.

### 4.8. Statistical Analysis

Statistical analyses were conducted using GraphPad Prism version 5.0 (GraphPad Software, Inc., La Jolla, CA, USA). The comparison of *STC2* expression between tumor and normal tissues was performed using Student’s *t*-test. Correlations between *STC2* expression and patient survival were analyzed using Spearman’s rank correlation test, a non-parametric approach suitable for assessing relationships between non-normally distributed variables. Kaplan–Meier survival curves were statistically analyzed using the log-rank test, which is appropriate for comparing survival outcomes between two groups. Statistical significance was set at *p* < 0.05, with thresholds for significance noted as * *p* < 0.05, ** *p* < 0.01, *** *p* < 0.001.

## 5. Conclusions

This study demonstrates that *STC2* plays a significant role in the progression of ER-positive breast cancer and may serve as a reliable marker for predicting both survival outcomes and positive responses to chemotherapy, particularly in grade II ER-positive breast cancer patients.

## Data Availability

The data supporting the findings of this study are publicly available. The transcriptomic data analyzed in this study were obtained from the GEO under the following accession numbers: GSE244574 [29], GSE202536 [30] and GSE GSE240671 [31]. These datasets provide information on breast cancer cell lines treated with Dox and other chemotherapeutic agents. All datasets can be accessed through the GEO database (https://www.ncbi.nlm.nih.gov/geo/; accessed on 15 August 2024).

## Supplementary Materials

The following supporting information can be downloaded at: https://www.mdpi.com/article/10.3390/ph18020235/s1, Figure S1: *STC2* expression across various cancer types compared to normal tissues; Figure S2: Bar graph showing the percentage of tumors with *STC2* expression higher than different cut-offs in normal tissues, along with specificity data; Figure S3: *STC2* mRNA expression and genetic alterations across various cancer types from the TCGA Pan-Cancer Atlas; Figure S4: *STC2* expression levels in breast cancer cell lines treated with Dox compared to control groups across three datasets; Figure S5: *STC2* gene expression levels before and after neoadjuvant chemotherapy in breast cancer patients.
