# Supplementary material for: Predictive Value of STC2 Gene Expression in Chemotherapy Response in Breast Cancer
Source: Pharmaceuticals (Basel). 2025 Feb 8;18(2):235. doi: 10.3390/ph18020235 (PMC11859796; doi:10.3390/ph18020235)
Supplement: Supplementary file 1 [file pharmaceuticals-18-00235-s001.zip › pharmaceuticals-3251529-supplementary.pdf]

Figure S1

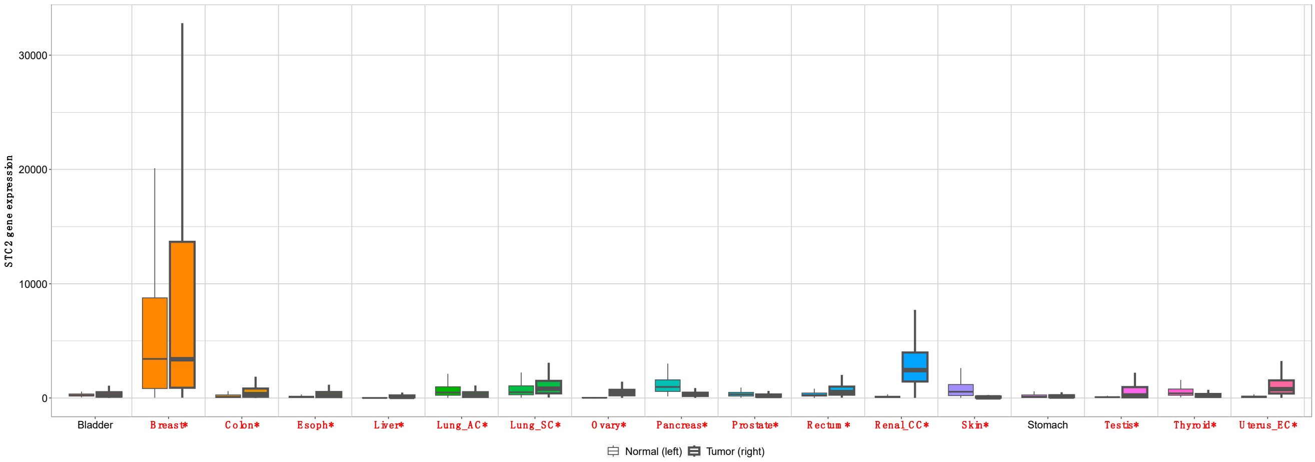

Figure S1. *STC2* expression across various cancer types compared to normal tissues. Box plots represent *STC2* expression levels in normal (left) and tumor (right) tissues from TCGA for different cancer types, including bladder, breast, colon, esophagus, liver, lung adenocarcinoma (Lung_AC), lung squamous cell carcinoma (Lung_SC), ovary, pancreas, prostate, rectum, renal clear cell carcinoma (Renal_CC), skin, stomach, testis, thyroid, and uterine endometrial carcinoma (Uterus_EC). Asterisks denote statistically significant differences between tumor and normal tissues. Data retrieved by TNM plot web server.

Figure S2

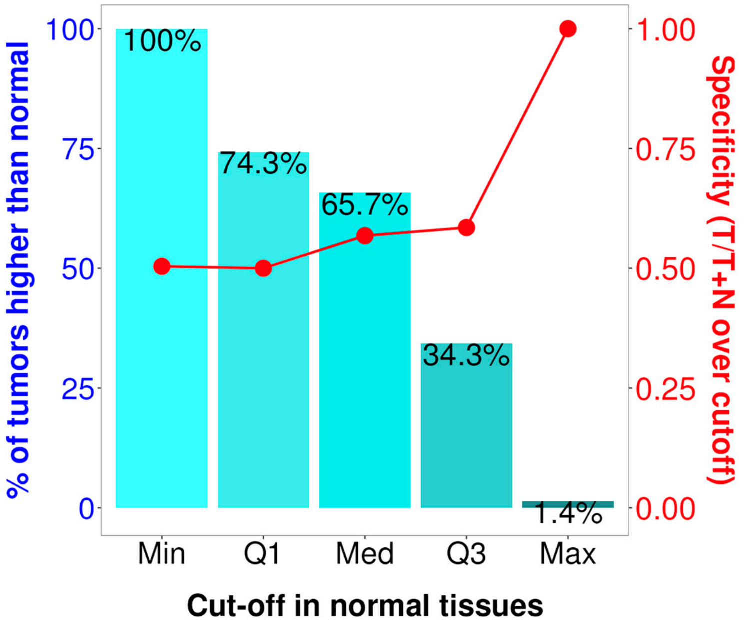

Figure S2. Bar graph showing the percentage of tumors with *STC2* expression higher than different cut-offs in normal tissues, along with specificity data. Plots consider paired tumor and adjacent normal tissues. Data from TNMplot web server.

Figure S3

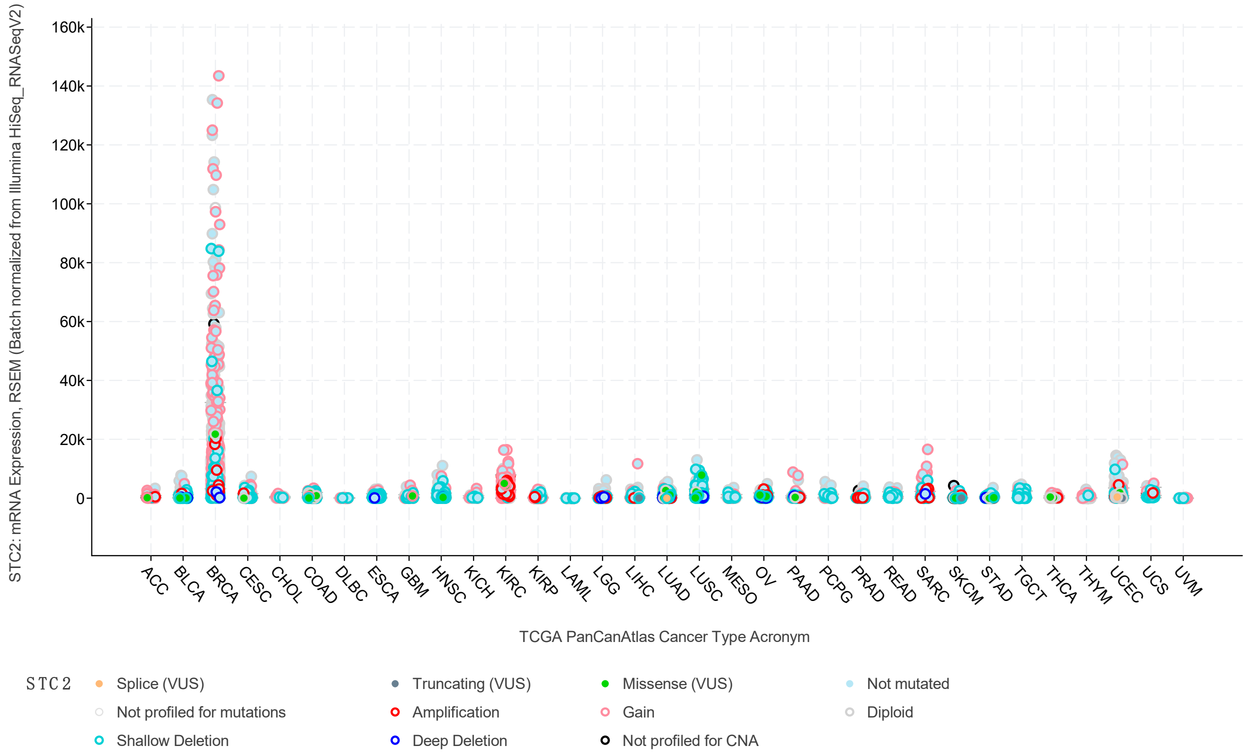

Figure S3. *STC2* mRNA expression and genetic alterations across various cancer types from the TCGA Pan-Cancer Atlas. The bubble plot shows the distribution of STC2 expression levels and associated genomic alterations across different cancer types. Each dot represents an individual sample, with colors indicating the type of mutation or copy number alteration. Data source: cBioPortal (RNA-seq data normalized from the TCGA Pan-Cancer Atlas).

Figure S4

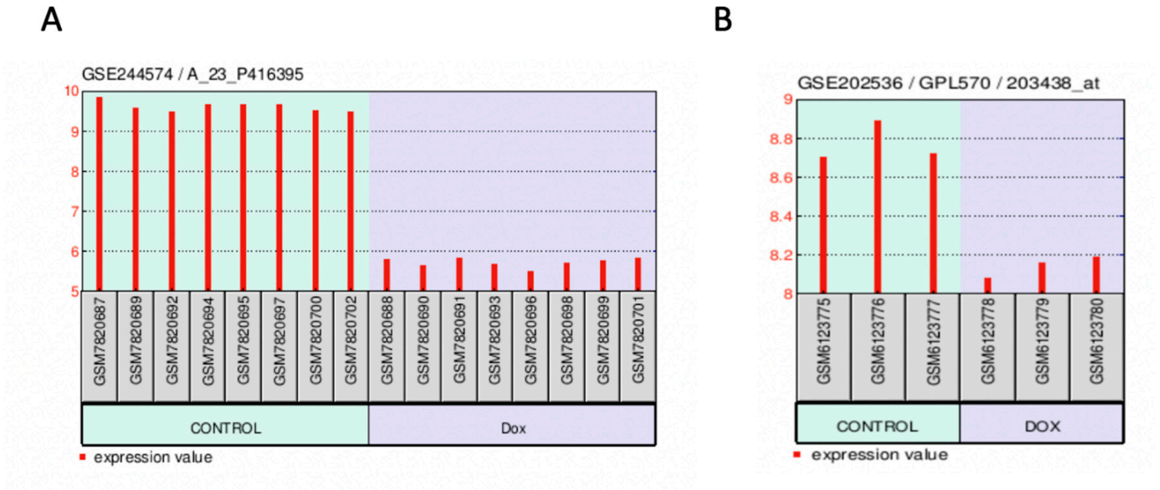

Figure S4. *STC2* expression levels in breast cancer cell lines treated with Dox compared to control groups across three datasets. The graphs display *STC2* expression values for each sample within the datasets: GSE244574 (**A**), GSE202536 (**B**). Expression values are indicated by red bars, highlighting the differential expression of *STC2* between control and Dox-treated conditions.

Figure S5

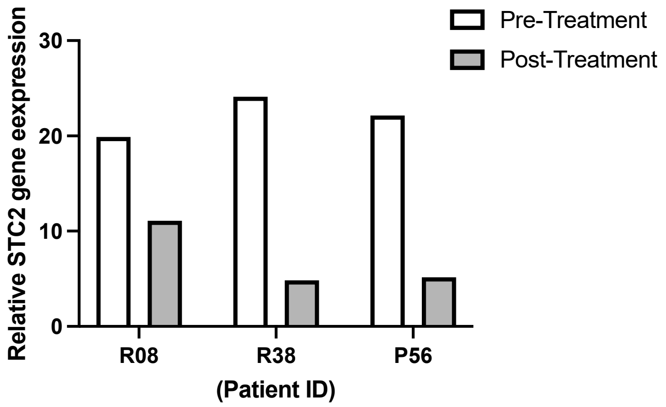

Figure S5. *STC2* gene expression levels before and after neoadjuvant chemotherapy in breast cancer patients. Data is shown for three patients (R08, R38, P56) from the GSE240671 dataset, all of whom underwent chemotherapy and had residual tumors post-treatment.
